# Evaluation of Pre-Analytical Performance Using IFCC Quality Indicators, Six Sigma Metrics and Root Cause Analysis in a Biochemistry Laboratory—A Retrospective Study

**DOI:** 10.3390/diagnostics16152357

**Published:** 2026-07-27

**Authors:** Soha Abdulrahman Alonaizan, Nadiah A. Alenaizan, Abdulwahab Z. Binjomah, Salman Aldosari, Shoukat Ali Arain

**Affiliations:** 1 College of Medicine, Alfaisal University, Riyadh 11533, Saudi Arabia; saalonaizan@alfaisal.edu (S.A.A.);; 2 Security Forces Hospital, Riyadh 11481, Saudi Arabia; alenaizan77@gmail.com

**Keywords:** pre-analytical errors, hemolysis, quality indicators, Six Sigma, root cause analysis, specimen rejection, laboratory medicine, specimen handling

## Abstract

**Background:** Pre-analytical errors account for the majority of failures across the total testing process. This study evaluated pre-analytical performance in a tertiary-care biochemistry laboratory in Riyadh, Saudi Arabia, using IFCC-aligned Quality Indicators (QIs), Six Sigma metrics, and structured Root Cause Analysis (RCA). **Methods:** A retrospective analysis of all biochemistry tests processed between January and December 2024 at a tertiary-care hospital was conducted. Rejected tests were classified into seven IFCC-aligned QI categories. Sigma metrics assessed process capability, Pareto analysis identified the vital few contributors, and RCA using the Ishikawa framework identified human, equipment, environmental, and process-related factors. Rejection patterns were described by department and work shift. **Results:** Of 845,647 tests performed, 10,783 (1.28%) were rejected, yielding an overall process capability of 3.89σ (Minimum Acceptable). Hemolysis was the leading cause (8186 tests; 75.92%) at 3.97σ, the only indicator classified as High against the IFCC WG-LEPS registry. The remaining six indicators demonstrated Good to Very Good performance (4.59σ–5.33σ). Pareto analysis identified hemolysis and inappropriate tube use (7.60%) as the vital few, jointly responsible for 83.52% of rejections. RCA implicated venipuncture technique, needle gauge selection, workload pressure, and prolonged tourniquet application as key contributors. The Emergency Department generated the highest inpatient rejection burden (38.7%). **Conclusions:** Although the overall rejection rate compared favorably with international benchmarks, hemolysis was the principal process vulnerability, with inappropriate tube selection as a secondary target. Recommended quality improvement strategies include structured phlebotomy training, real-time hemolysis index feedback, and Emergency Department-specific initiatives; structural solutions such as dedicated inpatient phlebotomy services warrant prospective evaluation alongside training-based interventions.

## 1. Introduction

Patient safety and systematic quality improvement form the cornerstone of modern healthcare delivery. Clinical laboratories serve as a central engine for clinical decision-making, and any variance in the integrity of laboratory outputs can lead to serious clinical repercussions, including delayed diagnoses, unnecessary secondary testing, inappropriate therapeutic interventions, and increased hospital lengths of stay [1,2].

Despite advancements in total laboratory automation, high-throughput analytical platforms, and advanced computing, the total elimination of laboratory errors remains elusive [3]. Contemporary laboratory medicine divides the total testing process into three distinct phases: pre-analytical, analytical, and post-analytical. While automated internal quality control software and external quality assessment schemes have driven analytical error rates to historical lows, the pre-analytical phase remains highly susceptible to error and variance [4].

The pre-analytical phase encompasses a multi-step sequence beginning with the clinical generation of a test order, spanning patient identification, specimen collection, tube labeling, sample transport, and initial processing within the laboratory. Because this phase is heavily decentralized and reliant on a heterogeneous cadre of healthcare professionals—including physicians, nurses, ward clerks, and couriers—it accounts for 60% to 70% of all laboratory errors documented across global literature. Specimen rejection due to pre-analytical non-conformities stands out as a leading driver of diagnostic inefficiency [5].

A recent global systematic review and meta-analysis of more than 16 million blood specimens established a pooled rejection prevalence of 1.99%, with clotted specimens (32.23%) and hemolysis (22.87%) representing the two leading causes worldwide [6]. High-acuity units, most notably intensive care units (ICUs) and emergency departments (EDs), consistently display significantly higher pre-analytical defect rates compared to outpatient phlebotomy centers [3,7].

The International Federation of Clinical Chemistry and Laboratory Medicine (IFCC) Working Group on Laboratory Errors and Patient Safety (WG-LEPS) formalized a series of harmonized Quality Indicators (QIs) to enable laboratories to systematically track and benchmark pre-analytical defect rates against international cohorts [2,4,8]. Modern quality systems increasingly pair QI monitoring with Six Sigma methodology to provide a normalized, internationally comparable measure of process capability [9], and with Root Cause Analysis (RCA) to identify the underlying human, equipment, environmental, and process factors driving errors—since QIs and Sigma metrics alone identify where and how often failures occur, but cannot explain why [10]. The combination of these three approaches enables laboratories to both quantify performance and design targeted, systemic interventions.

Within Saudi Arabia, the healthcare transformation under Vision 2030 has placed considerable emphasis on clinical standardization, institutional accreditation, and data-driven patient safety. Several regional studies have investigated pre-analytical error frequencies in Saudi clinical laboratories; however, their methodological scope has been limited. Alcantara et al. [3] and Alenezy et al. [11] reported rejection percentages and error-type distributions but did not convert these to normalized process capability metrics. Alshaghdali et al. [12] applied IFCC QIs and Sigma analysis in a hematology laboratory but did not include Pareto prioritization or structured root cause investigation. Iqbal et al. [13] described preanalytical error frequencies in a Saudi hematology setting without Sigma or systematic RCA. In addition, no published study from Saudi Arabia has evaluated a biochemistry-specific laboratory using the complete framework of IFCC WG-LEPS QIs, Six Sigma process capability metrics, Pareto analysis, and Ishikawa RCA within a single high-volume dataset.

This methodological gap matters for two practical reasons. First, reporting rejection rates as simple percentages, without Sigma conversion, prevents meaningful international benchmarking against the IFCC WG-LEPS registry and obscures the distinction between statistically stable processes and those at genuine risk of deterioration. Second, identifying which errors account for the majority of variance (Pareto) and investigating why they occur (RCA) are complementary steps that together enable targeted, sustainable intervention—yet the two have rarely been applied jointly in the regional literature. By integrating all four approaches within a large, 12-month biochemistry dataset from a tertiary-care hospital in Riyadh, this study aims to fill this evidence gap and provide a replicable quality evaluation model applicable to comparable institutions in the Gulf region.

## 2. Materials and Methods

### 2.1. Study Design and Setting

This retrospective observational study was conducted at Security Forces Hospital (SFH), a 500-bed tertiary care referral center in Riyadh, Saudi Arabia, from January to December 2024. All blood samples submitted for biochemistry testing during the study period were included, with each requested test set treated as an independent rejection opportunity. Non-blood specimens (e.g., urine, cerebrospinal fluid) and samples submitted to departments other than biochemistry were excluded.

### 2.2. Data Collection and Classification

A single blood collection event (specimen) may generate multiple test set requests within the same requisition, each of which can be independently flagged for a pre-analytical defect by the laboratory information system (LIS). The denominator used throughout this study represents the total number of test sets processed, and the numerator represents the total number of test-set rejection records as logged by the LIS—not unique physical specimens. Specimens were classified as originating from inpatient (IPD) or outpatient (OPD) departments based on the recorded patient location field.

### 2.3. Quality Indicator Analysis

For all seven QIs, a single uniform denominator—the total number of test sets received during the study period (*n* = 845,647)—was applied consistently for both percentage rejection rate and DPMO calculation, in accordance with the IFCC WG-LEPS convention of using total received requests as the common denominator across pre-analytical quality indicators [4] (Supplementary Table S1).

### 2.4. Six Sigma Analysis

Defects Per Million Opportunities (DPMO) values were calculated using the formula: DPMO = (Number of defects × 1,000,000)/Total number of opportunities (test sets). As stated above, one pre-analytical quality opportunity was defined as one test set—a discrete group of analytical requests processed together as a single unit by the LIS, which may encompass one or more individual analytes. One opportunity is therefore neither equivalent to one individual analyte, nor to one physical blood tube, nor to one laboratory requisition, but rather to the operational processing unit at which the LIS records and flags pre-analytical rejection events.

The IFCC WG-LEPS formulae specify “total number of samples” (physical specimens) as the denominator [4]. The use of test sets here represents a deviation from this strict definition; however, as the same test-set multiplier applies to both numerator and denominator, the practical impact on rejection rates is expected to be small, provided the ratio of test sets per specimen is approximately uniform across rejected and non-rejected specimens. Residual bias and the arithmetic consequence of alternative opportunity definitions are discussed in the Limitations section.

Corresponding Sigma (σ) values were derived by reference to the Westgard Six Sigma conversion table [9], assuming a 1.5σ long-term process shift. Performance tiers were classified as: very good (≥5.0σ), good (4.0–<5.0σ), minimum acceptable (3.0–<4.0σ), or unacceptable (<3.0σ). The test-set-level definition is used consistently throughout this study and should be considered when comparing Sigma values with studies using unique specimen counts as their denominator.

### 2.5. Pareto Analysis

The seven QIs were ranked in descending order of rejection frequency and cumulative percentages calculated. Error categories whose cumulative contribution reached the standard 80% threshold were classified as the vital few, in accordance with the Pareto (80/20) principle.

### 2.6. Root Cause Analysis

RCA was conducted by a multidisciplinary panel comprising five members: the laboratory quality officer, two senior biochemists, a senior phlebotomist, and a nursing ward representative from the ED (the highest-burden inpatient unit). The RCA process followed a structured, sequential four-stage approach.

In the first stage, quantitative data from the Sigma and Pareto analyses were presented to the panel as the primary input for cause identification. The Pareto-confirmed vital few—hemolysis (QI-10; 75.92%) and inappropriate tube selection (QI-9; 7.60%)—were designated as the primary effect endpoints for investigation, ensuring that causal analysis was anchored in data-driven prioritization rather than subjective assumption.

In the second stage, a structured brainstorming session was conducted using the Ishikawa (fishbone) framework. Each panel member independently generated candidate causes across five pre-defined domains—Human/Personnel, Equipment/Hardware, Environmental Conditions, Process Workflow, and Organizational—without discussion, to minimize anchoring bias. Candidate causes were then presented sequentially and mapped onto a shared Ishikawa diagram. In the third stage, each candidate cause was evaluated against two criteria: (a) plausibility—whether a direct mechanistic pathway from the candidate cause to hemolysis or tube selection error could be articulated; and (b) evidence—whether the candidate cause was supported by at least one of three data sources: monthly rejection trend records from the LIS, incident reports submitted by collecting units during the study period, or direct observation findings from workflow mapping sessions conducted in the ED, medical wards, and the central phlebotomy service.

In the fourth stage, consensus was achieved using a modified nominal group technique. Each panel member independently ranked the candidate causes within each domain by perceived contribution to hemolysis burden, using a five-point scale (1 = unlikely contributor; 5 = primary driver). Rankings were then disclosed simultaneously and discussed. Causes receiving a mean score of ≥4.0 from all five panelists were classified as primary contributing factors and included in the final Ishikawa diagram. Where initial divergence existed, a structured discussion was held and a re-vote conducted; in all cases, consensus was reached within two voting rounds. No formal weighting was applied across domains; the four final domains reflected the natural clustering of the validated cause set rather than a pre-specified hierarchical model.

### 2.7. Statistical Analysis

Data cleaning and descriptive statistical analysis were performed using Microsoft Excel (Microsoft Corp., Redmond, WA, USA). Descriptive statistics, including frequencies and percentages, were used to characterize rejection patterns across departments, work shifts, and quality indicators. The primary analytical framework for evaluating laboratory performance was process capability assessment using IFCC WG-LEPS QIs benchmarked against published quality specifications, and Six Sigma metrics expressed as DPMO and Sigma levels. 95% confidence intervals for individual QI rejection rates were calculated using the Wilson score interval method.

Formal inferential statistics—including significance testing, confidence intervals, and rate ratios—were not calculated for subgroup comparisons (shifts and departments) for two reasons. First, subgroup-level test volume denominators were not verifiable in the available LIS dataset. Second, in the context of a large dataset where even trivial differences would achieve statistical significance, the IFCC benchmarking framework and Sigma tier classifications provide more clinically interpretable measures of performance: a shift from Minimum Acceptable (3.89σ) to Good (≥4.0σ), or from High to Optimal in the IFCC registry, represents a meaningful quality threshold that conveys actionable information beyond a p-value. Subgroup comparisons are therefore presented descriptively, and differences between shifts and departments should be interpreted as exploratory observations rather than statistically confirmed differences.

### 2.8. Ethical Considerations

Ethical approval was obtained from the Research Ethics Committee of Security Forces Hospital, Riyadh, Saudi Arabia (Approval No. H-01-R-069; approved 31 December 2024). Individual written informed consent was waived by the ethics committee given the retrospective, de-identified nature of the study. The study was conducted in accordance with the principles of the Declaration of Helsinki.

## 3. Results

### 3.1. Overall Pre-Analytical Rejection Rate

During the study period, the clinical biochemistry laboratory received blood specimens for 845,647 test sets, of which 10,783 tests were rejected, with an overall pre-analytical rejection rate of 1.28%. Of the rejected tests, IPD accounted for 7524 (69.78%) and OPD for 3259 (30.22%). The overall process capability demonstrated a DPMO of 12,751 with an overall σ of 3.89 (Minimum Acceptable).

The analysis across the seven IFCC WG-LEPS QIs revealed a Sigma performance spectrum ranging from 3.97σ to 5.33σ (Table 1). Hemolyzed specimens (QI-10) accounted for 8186 (75.92%) of all pre-analytical errors with a DPMO of 9680 and a Sigma level of 3.97σ, placing QI-10 as the sole indicator within the Minimum Acceptable performance tier. Against IFCC WG-LEPS benchmarks, the hemolysis rate was classified as High.

The remaining six indicators demonstrated markedly stronger process control. Inappropriate blood collection tubes (QI-9), clotted specimens (QI-11), insufficient specimen volume (QI-12), and damaged samples (QI-14) recorded Sigma values of 4.59σ, 4.65σ, 4.69σ, and 4.74σ, respectively, all within the Good tier and meeting the IFCC Optimal benchmark. The highest process capability was observed for improperly labeled specimens (QI-15) and lost or unreceived samples (QI-8), both achieving Very Good performance. Despite its strong Sigma performance, QI-8 recorded a rate of 0.013%, marginally exceeding the IFCC High threshold of 0.010%.

### 3.2. Distribution Across Work Shifts

Across all three operational shifts, the distribution of rejection types is reported as a proportion of total rejections within each shift, rather than as a shift-specific rejection rate, as total test volumes processed per shift were not independently verifiable from the available dataset. Absolute rejection counts were highest in the morning shift (*n* = 6010; 55.74% of total rejections). Within each shift, hemolysis (QI-10) dominated the rejection profile consistently: 74.6% in the morning, 78.6% during the evening, and 76.8% in the night shift (Table 2). The ranking hierarchy of secondary and tertiary defects remained largely mirrored throughout. Wrong tube selection (QI-9) was the second most frequent error across all shifts, uniformly followed by clotted specimens (QI-11) and insufficient volume (QI-12). Damaged samples (QI-14) were proportionally more common during the morning shift (4.6%) compared to the evening (3.4%) and night (3.6%).

### 3.3. Departmental Distribution of Inpatient Rejections

A location breakdown of the 7524 tests rejected within inpatient services is shown in Table 3. These figures represent the absolute number and proportion of total inpatient rejections originating from each department; they do not represent department-specific rejection rates, as total test volumes submitted per department were not available. With this caveat, the ED generated the largest absolute rejection burden (2913; 38.7% of all inpatient rejections), consistent with its role as the highest-volume and highest-acuity inpatient unit. Medical Ward 6A (M6A) was the second largest source (1168; 15.5%), followed by the Short Stay Ward (738; 9.8%). Medical wards generated a higher absolute rejection count than surgical units, though without departmental test volume data, this observation may reflect higher throughput rather than lower collection quality. The ICU and CCU accounted for 4.7% (352) and 2.1% (155) of rejections, respectively. The ICU and CCU accounted for 4.7% (352) and 2.1% (155) of rejections, respectively.

### 3.4. Pareto Analysis

**Figure 1:**
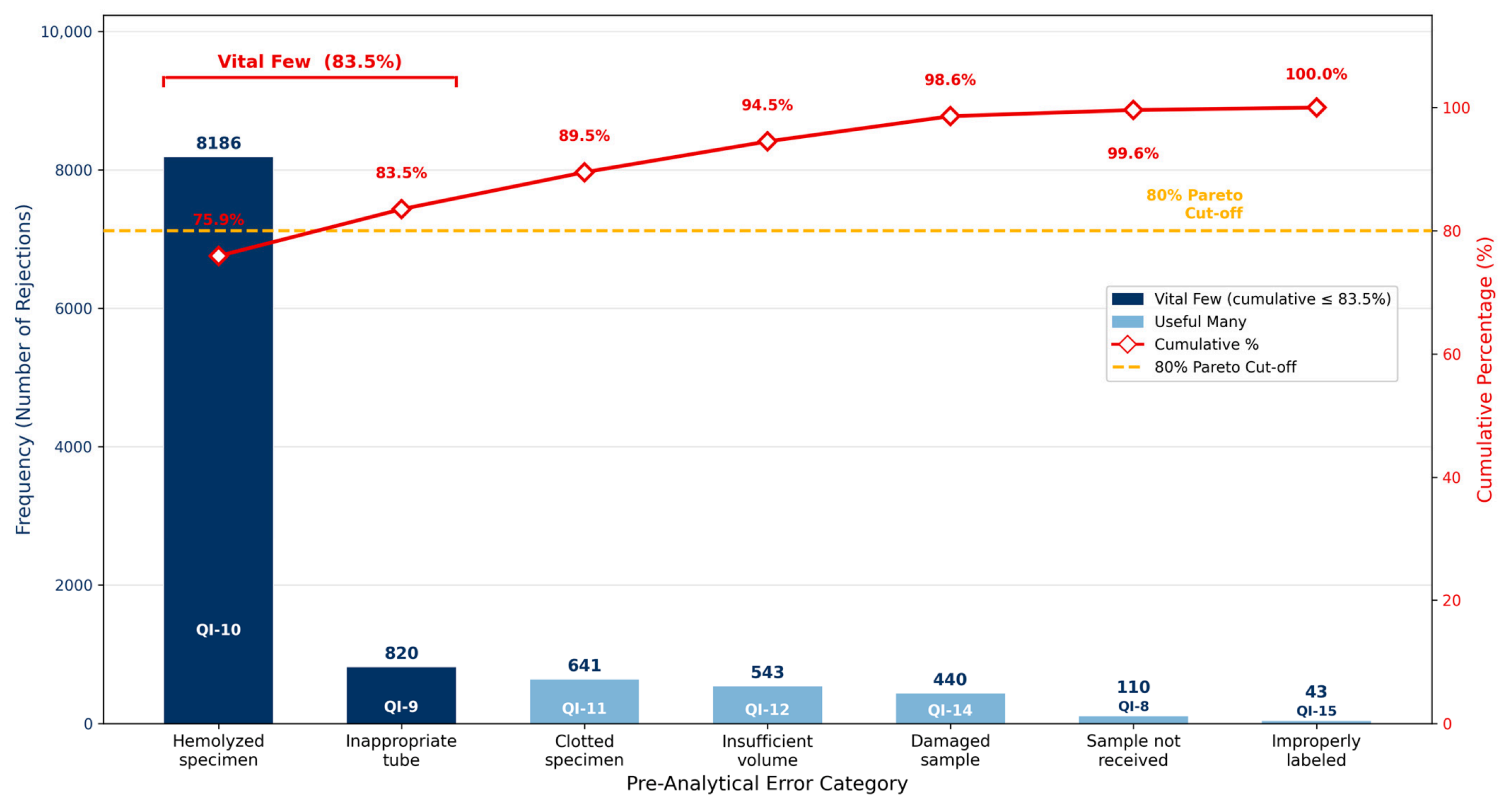
Pareto analysis of pre-analytical specimen rejections. Bars represent rejection frequency (left axis) and the line represents cumulative percentage (right axis). The dashed line indicates the 80% threshold. QI-10 (hemolysis) and QI-9 (wrong tube) constitute the vital few, jointly responsible for 83.52% of all rejections.

Pareto analysis of the 10,783 pre-analytical rejection events revealed a highly asymmetric error distribution, with two categories accounting for more than 80% of cumulative error (Figure 1). Hemolyzed specimens (QI-10) constituted the largest contributor (8186 rejections; 75.92%). The cumulative percentage crossed the 80% threshold upon addition of inappropriate blood collection tubes (QI-9; *n* = 820), bringing the cumulative total to 83.52%. The remaining five categories constituted the “useful many” (16.48%).

### 3.5. Root Cause Analysis

**Figure 2:**
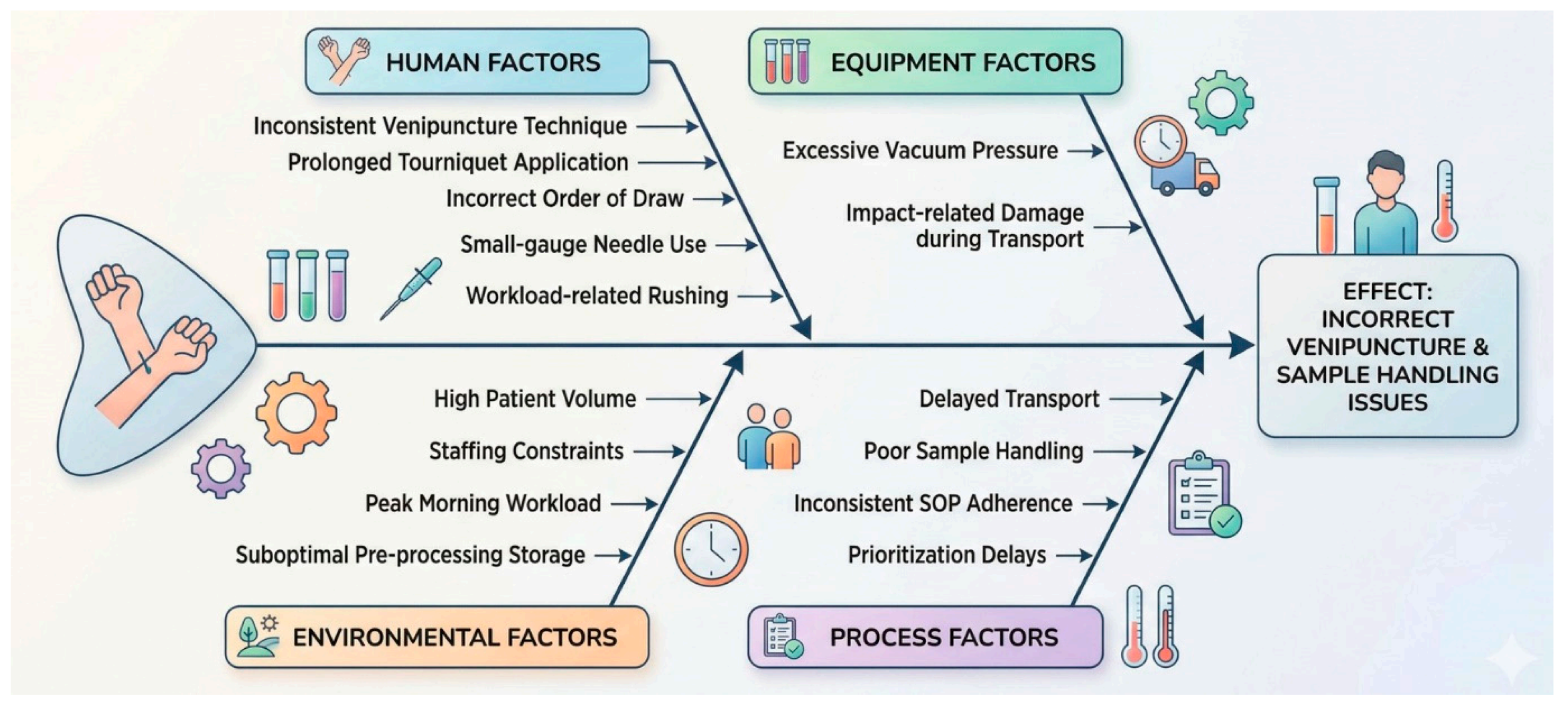
Ishikawa (fishbone) root cause analysis of pre-analytical specimen rejection at Security Forces Hospital, Riyadh, 2024. Causes are categorized across four operational domains: Human/Personnel, Equipment/Hardware, Environmental Conditions, and Process Workflow.

The multidisciplinary Ishikawa RCA (Figure 2) identified four principal domains of contributory factors. Human and personnel factors—including inconsistent venipuncture technique, inappropriate needle gauge selection (>23G), inadequate tube mixing, and training gaps among newly onboarded ward staff—constituted the largest category of modifiable causes. Equipment vulnerabilities, particularly excessive vacuum pressure during syringe transfer and mechanical trauma from improper butterfly needle use, represented additional technical targets. Environmental conditions were dominated by ED overcrowding, morning workload pressure, and night shift staffing constraints. Process workflow deviations, including prolonged tourniquet application, non-adherence to order-of-draw, and delayed specimen transport, completed the causal framework.

## 4. Discussion

During the 12-month study period, 10,783 tests (1.28%) were rejected, yielding a process capability of 3.89σ (DPMO: 12,751) within the Minimum Acceptable tier. Rejection rates varied from approximately 0.5% in North America to 2.8% in Asia, with a global pooled prevalence of 1.99% [6], placing SFH favorably within this distribution. However, the 3.89σ value reflects a lower boundary of acceptability, predominantly driven by hemolysis burden [2,14]. Disproportionate vulnerability to hemolysis is consistently reported where specimen collection is performed by personnel of varying training backgrounds [3].

Hemolyzed specimens (QI-10) recorded 3.97σ, the sole indicator below the Good threshold, and was classified as High against the IFCC WG-LEPS registry [2,8]. While hemolysis is widely cited as the leading cause of unsuitable specimens [14,15], this dominance is not universal: Cheng et al. [16] and Alenezy et al. [11] identified clotting as predominant in broader, multi-section laboratories, and the global meta-analysis by Getawa et al. [6] similarly found clotted specimens (32.23%) outranking hemolysis (22.87%). This suggests hemolysis dominance is more pronounced in biochemistry-exclusive testing streams, where it directly interferes with spectrophotometric assays [17].

Hemolyzed specimens (75.92%) and inappropriate tubes (7.60%) together accounted for 83.52% of all rejections, validating the Pareto 80/20 principle [18,19]. Comparable biochemistry-focused tertiary laboratories report similarly skewed distributions, in contrast to multi-section laboratories where clotting predominates [20,21]. QI-9 (4.59σ, DPMO 970), while within the Good tier and meeting the IFCC Optimal benchmark, remains a secondary correctable target through LIS-integrated electronic verification at order entry [22].

The Ishikawa RCA identified a multifactorial etiology spanning human, equipment, and process domains. Human factors—including technique variation, inappropriate small-gauge needle selection (>23G), inadequate tube mixing, and training gaps—emerged as the primary modifiable drivers, consistent with literature identifying technique-related variables as principal determinants of hemolysis [14,23]. A randomized trial confirmed manual aspiration substantially reduces hemolysis compared to vacuum-based collection in emergency settings [24]. At the process level, prolonged tourniquet application beyond the CLSI-recommended 60 s elevates lysis risk [25]; order-of-draw deviations introduce additive carryover risk [26]; and pre-centrifugation storage delays independently elevate hemolysis index over time [27].

Hemolysis dominated all three shifts uniformly, suggesting a systemic technique- and workflow-rooted failure rather than a shift-specific phenomenon. The morning shift carried the largest rejection volume (55.74%), consistent with peak throughput; the night shift’s marginally elevated hemolysis proportion (76.8%) may be consistent with reduced overnight staffing levels and the collection of specimens from more acutely unwell inpatients, though the present study does not include direct data on staffing ratios or patient acuity to confirm this relationship. Comparable shift-dependent patterns have been reported elsewhere, with out-of-hours collection associated with elevated unacceptable sample rates [28].

IPD generated 69.78% of rejections versus 30.22% from OPD. While the absence of denominator data prevents calculation of setting-specific rejection rates, this distribution may partly reflect differing collection environments and collector skill sets—including the use of dedicated trained phlebotomists in the OPD setting versus ward nursing staff in the IPD setting—a pattern that has been reported elsewhere [3,12], though direct confirmation from collector-level data was beyond the scope of this study. The ED alone generated 38.7% of IPD rejections and 27.0% of total rejections, consistent with international literature identifying the ED as the highest-risk pre-analytical environment, corroborated by a 141,609-encounter cohort identifying catheter gauge and insertion site as key hemolysis predictors [29]. Within the inpatient setting, medical wards generated a higher absolute rejection count than surgical units; however, as departmental test volume data were unavailable, this observation may reflect differences in throughput rather than differences in collection quality, and should be interpreted descriptively.

Based on the root cause domains identified, a multi-pronged quality improvement strategy addressing technique standardization, equipment optimization, workflow redesign, and competency monitoring may offer the greatest potential for durable improvement, consistent with frameworks proposed in the literature [14,15]. Potential strategies worthy of prospective evaluation include structured phlebotomy training programs for ward nursing staff, with emphasis on needle gauge selection, tube inversion technique, and tourniquet management; implementation of real-time hemolysis index monitoring at specimen receipt with structured feedback to collecting units; and ED-specific pre-analytical quality initiatives targeting the highest-burden inpatient unit. Tube selection errors remain a secondary but accessible target through LIS-integrated electronic decision support at order entry.

However, while structured phlebotomy training represents a logical first-line strategy, the published evidence of its sustained effectiveness is modest. Studies report improvement in the months immediately following training, but long-term maintenance is inconsistent, particularly in high-turnover inpatient environments where newly onboarded staff continuously replace trained personnel [30,31]. This suggests that training alone may be insufficient, and that structural solutions—including the deployment of dedicated professional phlebotomists to high-burden inpatient units—warrant consideration alongside educational interventions. The differential rejection burden observed within this institution between OPD (30.22%), where a dedicated trained phlebotomy team operates, and IPD (69.78%), where ward nursing staff collect under acute clinical pressures, provides indirect institutional evidence in support of this approach. A formal prospective cost–benefit analysis of professional phlebotomy deployment at SFH is beyond the scope of this retrospective study but represents a high-priority direction for future quality improvement investigation. The economic cost of the 10,783 rejected test sets identified in this study—encompassing repeat collection, laboratory reprocessing, delayed clinical decisions, and potential extended inpatient stays—was not formally quantified; a prospective cost-of-poor-quality analysis would provide the institutional business case needed to support structural workforce investment.

Expressing defect rates as DPMO and Sigma against the IFCC WG-LEPS registry provides an internationally comparable performance language for internal tracking and peer benchmarking [2,18]. Annual replication is recommended to monitor trajectories and detect deterioration early.

Several limitations of this study warrant acknowledgement. First, the single-institution, retrospective design precludes causal inference and limits generalizability; associations between shifts, departments, and rejection patterns are descriptive and subject to unmeasured confounding. Second, subgroup-level test volume denominators for individual shifts and departments were not available, precluding subgroup-specific rejection rate and Sigma calculations; the reported proportional analyses reflect absolute rejection counts and may in part reflect workload differences rather than true differences in collection performance. Future studies should capture shift-level and department-level test volumes to enable rate-based subgroup analysis. Third, the unit of analysis is the LIS test-set record rather than the unique physical specimen. The test-set-level definition is used consistently throughout and should be considered when comparing our Sigma values with studies using specimen-level denominators. However, since the same test-set multiplier applies to both numerator and denominator, the practical impact on the reported rejection rates is expected to be small, provided the ratio of test sets per specimen is approximately uniform across rejected and non-rejected specimens.

Two further methodological considerations apply to the IFCC WG-LEPS framework. First, quality specification threshold values were sourced from published IFCC WG-LEPS documents; direct verification against the live IFCC MQI platform (www.ifcc-mqi.com) was not possible as it was inaccessible, being subscription-based, and threshold values are periodically updated. Second, the official IFCC WG-LEPS formulae specify QI-specific denominators for certain indicators—notably total samples checked for hemolysis (QI-10) and total transported samples (QI-14)—which were not separately available from the LIS dataset; a single global denominator was applied to all QIs, which may underestimate the true error rates for these specific indicators and limit direct comparability with IFCC registry values derived from QI-specific denominators. Regarding the RCA, the multidisciplinary panel did not include hospital administrators, whose engagement is essential for translating findings into structural policy change—particularly for workforce solutions such as dedicated inpatient phlebotomy teams; broader nursing staff perspectives on feasible solutions were also not formally solicited. Finally, the economic cost of pre-analytical rejections was not quantified; a prospective cost-of-poor-quality analysis is recommended to provide the institutional business case for structural quality investment.

## 5. Conclusions

This study provides a comprehensive, metrics-driven evaluation of pre-analytical quality performance in a tertiary-care biochemistry laboratory over 12 months. Analysis of 10,783 rejection records across 845,647 processed tests revealed an overall process capability of 3.89σ (Minimum Acceptable), favorably positioned within the global pooled rejection prevalence. Hemolyzed specimens were the dominant and sole sub-threshold cause (75.92%; 3.97σ), and together with inappropriate tube selection constituted the Pareto vital few, jointly responsible for 83.52% of total pre-analytical variance. Root cause analysis implicated modifiable human, equipment, and process workflow factors—most notably venipuncture technique, needle gauge selection, and tourniquet management—as the primary drivers of hemolysis. Departmental mapping identified the Emergency Department as the highest-burden inpatient unit (38.7% of IPD rejections).

Structured phlebotomy training for ward nursing staff, real-time hemolysis index feedback, and ED-specific quality initiatives represent the highest-priority targets for prospective quality improvement programs, based on the Pareto prioritization and root cause findings of this study. The IFCC WG-LEPS quality indicator framework applied alongside Six Sigma methodology provides an internationally benchmarked performance language supporting both longitudinal internal monitoring and peer-institution comparison.

## Figures and Tables

**Table 1:** Six Sigma performance analysis and IFCC benchmarking of pre-analytical quality indicators (QIs).

QI	Error Type	*n*	Rate % (95% CI)	DPMO	σ	Performance ^a^	IFCC Level
QI-10	Hemolyzed specimen	8186	0.968 (0.947–0.989)	9680	3.97	Min. Acceptable	High
QI-9	Wrong tube	820	0.097 (0.091–0.104)	970	4.59	Good	Optimal
QI-11	Clotted specimen	641	0.076 (0.070–0.082)	758	4.65	Good	Optimal
QI-12	Insufficient volume	543	0.064 (0.059–0.070)	642	4.69	Good	Optimal
QI-14	Damaged sample	440	0.052 (0.047–0.057)	520	4.74	Good	Optimal
QI-8	Sample not received	110	0.013 (0.011–0.016)	130	5.04	Very Good	Below Min. ^b^
QI-15	Unlabeled specimen	43	0.005 (0.004–0.007)	51	5.33	Very Good	Optimal

DPMO, defects per million opportunities; QI, quality indicator; CI, confidence interval. DPMO and Sigma (σ) calculated using 845,647 total received test sets. Rate % = (*n*/845,647) × 100. 95% CI calculated using the Wilson score interval method. ^a^ Westgard performance classification (1.5σ long-term process shift): Very Good ≥5.0σ; Good 4.0–<5.0σ; Minimum Acceptable 3.0–<4.0σ; Unacceptable <3.0σ. ^b^ Despite Very Good Sigma (σ 5.04), rate of 0.013% marginally exceeds the IFCC High threshold of 0.010. Overall rejection rate: 1.275% (95% CI: 1.251–1.299%), *n* = 10,783/845,647.

**Table 2:** Distribution of pre-analytical error types across work shifts.

QI	Error Type	Morning (*n* = 6010)	Evening (*n* = 2243)	Night (*n* = 2530)
QI-10	Hemolyzed specimen	4482 (74.6%)	1762 (78.6%)	1942 (76.8%)
QI-9	Wrong tube	464 (7.7%)	158 (7.0%)	198 (7.8%)
QI-11	Clotted specimen	368 (6.1%)	131 (5.8%)	142 (5.6%)
QI-12	Insufficient volume	316 (5.3%)	95 (4.2%)	132 (5.2%)
QI-14	Damaged sample	274 (4.6%)	76 (3.4%)	90 (3.6%)
QI-8	Sample not received	77 (1.3%)	16 (0.7%)	17 (0.7%)
QI-15	Unlabeled specimen	29 (0.5%)	5 (0.2%)	9 (0.4%)

**Table 3:** Pre-analytical rejection distribution across the top 10 inpatient (IPD) clinical locations (*n* = 7524).

Rank	Department	*n*	%
1	Emergency Department	2913	38.7
2	Medical Ward 6A	1168	15.5
3	Short Stay Ward	738	9.8
4	Medical Ward 4B	659	8.8
5	Surgical Ward 5B	393	5.2
6	ICU	352	4.7
7	Surgical Ward 5C	217	2.9
8	Surgical Ward 5A	172	2.3
9	CCU	155	2.1
10	Newborn ICU (NICU)	117	1.6

## Data Availability

The data that support the findings of this study are available from Security Forces Hospital, Riyadh, Saudi Arabia, but restrictions apply to their availability. Data are available from the corresponding author upon request.

## Supplementary Materials

The following supporting information can be downloaded at: https://www.mdpi.com/article/10.3390/diagnostics16152357/s1, Table S1: IFCC WG-LEPS Quality Indicators: official formulae and definitions applied in this study.
