# Supplementary material for: Evaluation of Pre-Analytical Performance Using IFCC Quality Indicators, Six Sigma Metrics and Root Cause Analysis in a Biochemistry Laboratory—A Retrospective Study
Source: Diagnostics (Basel). 2026 Jul 27;16(15):2357. doi: 10.3390/diagnostics16152357 (PMC13464699; doi:10.3390/diagnostics16152357)
Supplement: Supplementary file 1 [file diagnostics-16-02357-s001.zip › diagnostics-4436661-supplementary.pdf]

Supplementary Table S1

Table S1. IFCC WG-LEPS Quality Indicators: official formulae and definitions applied in this study.

QI Code	IFCC MQI Label	Error Category	Official Formula [a]	Definition
**QI-10** [b]	Samples hemolyzed	Hemolyzed specimen	% of (Number of samples with free Hb > 0.5 g/L / Total number of checked samples) × 100	Any sample where one or more tests were not performed, or results were rejected or not reported due to hemolysis
**QI-9**	Incorrect sample type — wrong container (Pre-WroCo)	Wrong collection tube	% of (Number of samples collected in wrong containers / Total number of samples) × 100	Specimen collected in the wrong tube type, e.g., PT test collected in an EDTA tube
**QI-11**	Samples clotted (Pre-Clot)	Clotted specimen	% of (Number of samples clotted / Total number of samples with an anticoagulant) × 100	e.g., clotted FBC tubes
**QI-12**	Incorrect fill level — insufficient volume (Pre-InsV)	Insufficient sample volume	% of (Number of samples with insufficient sample volume / Total number of samples) × 100	Tubes or syringes not filled to correct level
**QI-14** [b]	Unsuitable samples — damaged during transport (Pre-DamS)	Damaged sample	% of (Number of samples damaged during transportation / Total number of samples) × 100	Sample not stored or transported correctly
**QI-8**	Unsuitable samples — not received (Pre-NotRec)	Sample not received	% of (Number of samples not received / Total number of samples) × 100	Sample ordered but never received by the laboratory
**QI-15**	Misidentification errors — unlabeled samples (Pre-MisS)	Unlabeled/misidentified specimen	% of (Number of unlabeled samples / Total number of samples) × 100	Samples with fewer than 2 identifiers or unlabeled

[a] Formulae and definitions reproduced from Plebani et al. (2014) [4]. The official IFCC WG-LEPS formulae specify "total number of samples" (physical specimens) as the denominator; in this study, a single uniform denominator — total test sets processed (n = 845,647) — was applied to all QIs, as rejection records were captured at the test-set level by the LIS.

[b] The official IFCC WG-LEPS formulae specify QI-specific denominators for QI-10 (total samples checked for hemolysis) and QI-14 (total transported samples), which were not separately available from the LIS dataset. The global denominator was applied to these QIs, which may underestimate the true error rates for these indicators relative to values calculated using QI-specific denominators.
